# An FPT Approach for Predicting Protein Localization from Yeast Genomic Data

**DOI:** 10.1371/journal.pone.0014449

**Published:** 2011-01-19

**Authors:** Jin Wang, Chunhe Li, Erkang Wang, Xidi Wang

**Affiliations:** 1 State Key Laboratory of Electroanalytical Chemistry, Changchun Institute of Applied Chemistry, Chinese Academy of Sciences, Changchun, Jilin, China; 2 Departments of Chemistry, Physics and Astronomy, State University of New York at Stony Brook, Stony Brook, New York, United States of America; 3 Graduate School of the Chinese Academy of Sciences, Beijing, China; 4 Citibank, Sao Paulo, Brazil; Michigan State University, United States of America

## Abstract

Accurately predicting the localization of proteins is of paramount importance in the quest to determine their respective functions within the cellular compartment. Because of the continuous and rapid progress in the fields of genomics and proteomics, more data are available now than ever before. Coincidentally, data mining methods been developed and refined in order to handle this experimental windfall, thus allowing the scientific community to quantitatively address long-standing questions such as that of protein localization. Here, we develop a frequent pattern tree (FPT) approach to generate a minimum set of rules (mFPT) for predicting protein localization. We acquire a series of rules according to the features of yeast genomic data. The mFPT prediction accuracy is benchmarked against other commonly used methods such as Bayesian networks and logistic regression under various statistical measures. Our results show that mFPT gave better performance than other approaches in predicting protein localization. Meanwhile, setting 0.65 as the minimum hit-rate, we obtained 138 proteins that mFPT predicted differently than the simple naive bayesian method (SNB). In our analysis of these 138 proteins, we present novel predictions for the location for 17 proteins, which currently do not have any defined localization. These predictions can serve as putative annotations and should provide preliminary clues for experimentalists. We also compared our predictions against the eukaryotic subcellular localization database and related predictions by others on protein localization. Our method is quite generalized and can thus be applied to discover the underlying rules for protein-protein interactions, genomic interactions, and structure-function relationships, as well as those of other fields of research.

## Introduction

Over the past decade, progress in the fields of proteomics has been both rapid and extensive. However, many fundamental proteomic data sets remain poorly comprehended, including those built to derive the subcellular localization of proteins. Subcellular localization, is a basic feature of proteins, underlying the mechanism by which cells classify newly synthesized proteins and send them off to their final destinations [1]–[3].

The prediction of protein localization is paramount in the pursuit to learn the function and role of proteins involved in all cellular processes [4]. Localization data can also be used to evaluate protein information indicated from genetic data [3], [5]. Additionally, the subcellular localization of a protein can be used to guide predictions about its mechanism of action [3], [6], [7]. Addtionally, one can gain inference on which pathway an enzyme belongs to with the knowledge of its proper sucellular localization and basic function in hand [8].

Various methods have been developed to predict the subcellular localization of proteins. An integrated expert system was developed to sort proteins into different compartments using sequentially applied “if-then” rules [9], [10]. This method has the advantage that it could potentially mimic the actual physical decisions in the underlying biological classification process. Another more probabilistic approach [11], [12] was developed, using a "k nearest neighbors" method to classify proteins according to the localization of their closest relatives. Additionally, some approaches related to sequence composition are adapted to predict subcellular localization [1]. For instance, a method combining overall composition with neural networks [8] has been used to sort proteins directly into different compartments, and Andrade et al. employed the composition of surface residues to predict subcellular localization [13]. Moreover, a database of protein subcellular localization has been presented [14], in which the authors annotated the entire proteomes of eukaryotic organisms using a hierarchical prediction method.

To predict subcellular localization correctly, one must integrate data from a multitude of sources. Progress has recently made [6], [15] via the combination of several attributes into one integrated predictor, exploiting the predictions of other methods in addtion to the raw data directly. Data mining methods can be used for feature integration, such as Bayesian network approaches [6],[16],[17], decision trees [18], support vector machines [19], and neural network [6], [20].

In this paper, we develop an adapted frequent pattern tree method (FPT) [21]–[23] to generate a minimum set of rules and apply it to integrate protein localization features of multiple data sources. Different protein localization features form different patterns. Accordingly, the number of possible patterns grows combinatorially with number of features. For a given database, FPT has the advantage because it exhaustively searches for interactive patterns among all possible components up to a specified minimum number of appearances within the database-support level. In order to get a better prediction of results, the support should be small, because it controls the degree of statistical robustness. When the support is set to be 1, FPT can find all interaction patterns within the development database, including those often missed by other statistical methods. FPT patterns can be considered as rules with attributes constructed by the protein localization features. We build all possible rules to predict protein locations in the form of: if feature-1 and feature-2, etc., then the corresponding location expected. Our objective is to predict which location proteins belong to given their features gathered from different sources. A problem of using FPT in practice, is that rules generated by FPT largely correlate or overlap with each other. We implemented the FPT algorithm to generate a minimum number of rules (mFPT) without losing detection accuracy.

The mFPT method can extract significant rare patterns from large amounts of data, and can thus discover the underlying rules and make predictions. It is a powerful method of data mining and can be used for scientific and engineering fields such as bioinformatics, drug discovery, chemometrics, engineering design and quality control, environmental control. It may also be used to improve administration of government and private corporations in sectors as diverse as health care, IRS, credit, database marketing, internet shopping, and customer relationship management, fraud detection, financial risk management, insurance etc.. [24]

Our study is motivated by an earlier integrated method for localizing yeast proteins using a Bayesian formalism. The authors of this method carefully constructed various sets of yeast proteins of known localization based on merging, filtering, and standardizing the annotations in MIPS(Munich Information Center for Protein Sequences) [25]–[27], Swiss-Prot and YPD(Yeast Protein Database) [28], and trained and tested this system against these sets [1].

We compare the predicted results with the actual results for the holdout sample for evaluating the prediction accuracy with several statistical measures and find that mFPT performs best in these data mining methods in predicting protein localization. Rules for predicting protein localization are built consequently as the results of frequent patterns. Furthermore, using these rules, we make the prediction of locations to the overall populations in the entire yeast genome including 4700 proteins without annotated locations in database. Setting 0.65 as the minimum hit-rate(ratio of positives over positives plus negatives) threshold, we obtained 138 proteins that mFPT predict differently from SNB(Simple naive bayesian method) [1]. By querying these 138 proteins against the database we derived novel predictions for the 17 proteins whose localization is heretofore unknown. These predictions can serve as putative annotations and should provide preliminary clues for experimentalists. In addition, we also compared our prediction results to those derived by other methodologies, setting 0.65 as the minimum hit-rate threshold against eSLDB(eukaryotic subcellular localization database) [14].

## Results

### The Datasets

The training and testing data for predicting protein localization are from Swiss-Prot [31] and MIPS [25], [26], and Yeast Protein Database [28]. One dataset of localized yeast proteins were prepared [1], called Localized-1342, included 1342 proteins. This dataset include 704 proteins in Swiss-Prot with high-quality localization, and 638 proteins with high-quality localization in MIPS that have low-quality in Swiss-Prot. Here, quality for each proteins represents the confidence level that a given protein belongs to one location, based on real experimental evidence.

The dataset Localized-1342 includes all proteins that have non-conflicting localizations in either MIPS, Swiss-Prot or both, as well as proteins that are not annotated to have a predicted localization. We therefore chose this dataset to predict and test the results [1].

The proteins in the Localized-1342 dataset are mainly classified into 12 sub-cellular compartments. The 12 compartments were collapsed into five new "generalized" compartments combining together related smaller compartments. The new compartments are the nucleus (N), mitochondria (M), cytoplasm (C), membrane (T), and secretory pathway (E for endoplasmic reticulum or extracellular). The T compartment included all the integral transmembrane (plasma membrane, cell membrane and membranes of various compartments such as mitochondria, nucleus, Golgi) proteins, whereas all the secreted proteins and proteins in the secretory pathway and small organelles (i.e. proteins in the Golgi, vacuoles, endoplasmic reticulum, vesicles and peroxisome) are classified to E compartment.

In order to predict localization using mFPT method, we needed to generalize the features under consideration. There were 19 genomic features [1] included in our calculation, and they were divided into three categories in terms of the information they were derived from: (1) motifs (12 features); (2) overall-sequence (2 features); and (3) whole-genome (5 features). Table 1 gives the description of 19 different features. For every feature, proteins were divided into definite numbers of bins in terms of the different feature values. Then we used numbers(from 1 to 113) to represent different bins for different features. For every feature, the biggest number represented no feature record for one protein, and the other numbers corresponded to different bins separately.

**Table 1:** Features description.

Feature	Type	Subtype	Bins(range)	Description
MIT1	Motif	Signal	2(1–3)	More than one N-terminal residue is cut (good chance of being mitochondrial) [28]
GLYC	Motif	Signal	11(4–14)	Number of predicted N-glycosylation sites (NXS/T) [10]
SIGNALP	Motif	Signal	2(15–17)	Secretory signal peptide according to the SignalP server [44]–[46]
SIG1	Motif	Signal	2(18–20)	If a protein has a signal sequence. The pattern consists of a charged residue within the first seven residues, followed by a stretch of 14 residues with an average GES hydrophobicity less than -1 kcal/mol [1]
NUC1	Motif	Signal	6(21–27)	Four-residue patterns of 1. All basic amino acid residues (K or R) or 2. Three basic amino acids (K or R), and one H or P [10]
PI	Overall-sequence	Isoelectric point	10(28–38)	pI (isoelectric point) values [25]–[28]
TMS1	Overall-sequence	Transmembrane helix	5(39–44)	Prediction results of whether a protein has transmembrane (TM) segments. TM segments were identified using the GES hydrophobicity scale [47]. The values from the scale for residues in a window of size 20 were averaged, and then compared against a cutoff value. Boyd and Beckwith MaxH criteria was used to set cutoffs as in previous analyses [48]–[50]
MAYOUNG	Whole-genome	Absolute expr.(GeneChip)	10(45–55)	Absolute mRNA expression in a GeneChip experiment [32]
KNOCKOUT	Whole-genome	Knockout mutation	2(56–58)	Knockout mutation (lethal or viable). [25]–[28]
MRDIASD	Whole-genome	Expr.fluctuation (Diauxic shift)	10(59–69)	Standard deviation in mRNA expression level over time (i.e. expression fluctuation) for a protein in the diauxic shift experiment [51]
PLMNEW1	Motif	Signal	2(70–72)	Plasma membrane signal [10]
FARN	Motif	Signal	2(73–75)	C-terminal farnesylation site: the sequence pattern consists of a cysteine followed by two aliphatic residues and one more residue at the C terminus [52]
GGSI	Motif	Signal	2(76–78)	C-terminal geranylgeranylation site [52]
MIT2	Motif	Signal	2(79–81)	Mitochondrial matrix import sequence: The N-terminal of the protein has repeated alternating hydrophobic and hydrophilic patterns, and the protein contains at least four S or T residues in its 20 N-terminal residues.
HDEL	Motif	Signal	2(82–84)	Endoplasmic reticulum retention signal (HDEL) [10]. We checked for the presence of this signal in the nine C-terminal residues
NUC2	Motif	Signal	3(85–88)	Pattern starting with a P and followed within three residues by a basic four-residue segment containing K or R residues [10]
POX1	Motif	Signal	2(89–91)	C-terminal peroxisome import signal ([SA](KRH]L) [10]
MRCYELU	Whole-genome	Expr.fluctuation (Cell cycle)	10(92–102)	Standard deviation in mRNA expression level over time (i.e. expression fluctuation) for a protein in the elutriation time series experiment in Yeast Cell Cycle Analysis Project [53]
MRCYCSD	Whole-genome	Expr.fluctuation (Cell cycle)	10 (103–113)	Standard deviation in mRNA expression level over time (i.e. expression fluctuation) for a protein in the alphafactor arrest time series experiment in Yeast Cell Cycle Analysis Project [53]

For every feature, proteins are divided into a definite number of bins in terms of the different feature values. Then we use number(from 1 to 113) to represent different bins for different features. For every feature, the biggest number represents no feature record for one protein, and the other numbers correspond to different bins separately. For example, for feature Mit1, 1 represents no More than one N-terminal residue is cut for one protein, 2 represents More than one N-terminal residue is cut for one protein, and 3 denotes no feature record for this protein.

The features in the motif class were based on a small sequence pattern in a protein. For example, the feature HDEL(the endoplasmic reticulum retention signal) represented the presence or absence of the HDEL motif at the C terminus of a protein, then we used numbers 82 to 84 to represent this feature, for which 82 denoted the absence of the HDEL motif, 83 denoted the presence of the HDEL motif, and 84 denoted no feature information for this protein.

The overall-sequence features were based on the entire sequence of a protein. For instance, the feature PI was the isoelectric point PI of a protein, while the feature TMS1 represented the number of predicted transmembrane segments in a protein. At last, the whole genome features were derived from whole-genome level data. For instance, the feature MAYOUNG contained the mRNA absolute expression data from the experiments of Young et al [32], and feature MAYOUNG were divided in to 10 bin(number 45 to 55), with 55 representing no feature record for one protein, and 44 to 54 corresponded to mRNA absolute expression data from low to high.

Input patterns for mFPT are shown in Table 2, in which each line represents a protein, and each column represents one of 19 genomic features, denoting separately MIT1, GLYC, SIGNALP, SIG1, NUC1, PI, TMS1, MAYOUNG, KNOCKOUT, MRDIASD, PLMNEW1, FARN, GGSI, MIT2, HDEL, NUC2, POX1, MRCYELU, MRCYCSD. The last column indicates the localization of protein, separately represented by 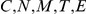. Localized-1342 included 1342 proteins, separately belonging to five different locations. To get an overall assessment of how the prediction performs, we divided the data samples into training (70%) and testing (30%) files.

**Table 2:** Input format of FPT.

Mt1	Gly	Sig	Sig1	NC1	PI	TMS	MAY	KNO	MRD	PLM	FAR	GGS	MT2	HDE	NC2	POX	MRC	MCC	Location
1	4	16	19	27	31	42	54	56	68	70	73	76	79	82	85	89	97	109	(E)
1	9	15	18	27	34	39	54	56	67	70	73	76	79	82	85	89	92	108	(C)
1	13	15	18	27	28	39	50	57	59	70	73	76	79	82	85	89	92	105	(N)
2	5	15	19	27	34	39	53	56	64	70	73	76	79	82	85	89	95	112	(M)
2	5	16	19	27	30	39	53	56	62	70	73	76	79	83	85	89	98	109	(E)

The meaning of category values are as Table 1.

### Rules of Predicting Protein Localization

We executed the mFPT algorithm with the following steps:

- Run FPT once, produce a complete set of patterns.
- Sort these rules according to their performances. (Here we used the product of hit-rate(ratio of positives over positives plus negatives) and square root of number of hits by the rule.)
- Select the best rule. (We chose highest hit-rate above support level.)
- Remove the samples hit by this rule, go to step 1 and run FPT again.

mFPT method prescribes that the lower the minimum support is, the more accurately the rules are predicted. In consideration of computing time cost, we chose the minimum support as 2, the minimum hit-rate as 0.5, and the rules below them were considered to be insignificant.

In the computation, we predicted five locations individually. After almost 50 loops, we got 45,31,17,34,15(total 142 rules) rules respectively for 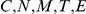 five locations, as is partly shown in Table 3 and entirely shown in File S1. At last, we integrated the five predicting results and obtained the final predictions for localization.

**Table 3:** Rules of FPT.

Hit-rate	Hit-number	Rules	Rules label	Location
0.852	163	76 89 1 54	C1	(C)
0.599	15	89 85 27 1 56 68	C2	(C)
0.603	222	73 76 82 89 79 15 1 57	N1	(N)
0.785	28	89 70 15 13	N2	(N)
0.747	91	73 85 15 27 2	M1	(M)
0.666	6	89 85 27 56 47 36	M2	(M)
0.916	12	89 85 1 56 43	T1	(T)
0.714	7	89 85 27 18 53 62	T2	(T)
0.789	19	89 85 27 2 19 16	E1	(E)
1	2	89 85 5 67 16	E2	(E)

The meaning of category values are as table 1.

Table 3 shows 10 best rules when the minimum hit-rate was set to 0.5, and Table 4 shows all the rules when the minimum hit-rate was set to 0.65.

**Table 4:** Rules of FPT used to predict Unknown-4700 with 0.65 as hit-rate threshold cut.

Hit-rate	Hit-number	Rules	Rules label	Location
0.852	163	76 89 1 54	(C1)	(C)
0.785	28	89 70 15 13	(N2)	(N)
0.666	18	73 82 89 79 15 1 11	(N3)	(N)
0.747	91	73 85 15 27 2	(M1)	(M)
0.916	12	89 85 1 56 43	(T1)	(T)
0.789	19	89 85 27 2 19 16	(E1)	(E)

The meaning of category values are as Table 1.

In Table 3 and Table 4, the first column represents the hit-rate of the corresponding patterns, and the second column denotes the number of hits (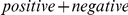). From Table 4, we were able to explore the meaning of each rule, and by checking the rules we discovered the 10 most important features affecting protein localization included MIT1, GLYC, MAYOUNG, GGSI, POX1, SIGNALP, PLMNEW1, TMS1, KNOCKOUT, NUC2. For these 10 ten features, MIT1, GGSI, SIGNALP were from amino acid sequence, GLYC, POX1, PLMNEW1, TMS1, NUC2 were predicted motifs, and only MAYOUN and KNOCKOUT 2 features were from other experiments. This showed that our predictor does not rely on the experiment information too heavily.

For these rules, the rule C1 states that when protein has no more than one N-terminal residue cut(feature MIT1 is 1), higher absolute mRNA expression value(feature MAYOUNG is 54), no C-terminal geranylgeranylation site(feature GGSI is 76) and no C-terminal peroxisome import signal(feature POX1 is 89), it has a higher probability of being in cytoplasmic location(C location).

Rule N2 denotes that when protein has no secretory signal peptide(feature SIGNALP is 15), more N-glycosylation sites(GLYC is 13), no plasma membrane signal(PLMNEW1 is 70) and no C-terminal peroxisome import signal(feature POX1 is 89), it has a higher probability of being localized to the nucleus(N location).

Rule M1 shows when protein has more than one N-terminal residue cut(MIT1 is 2), has no secretory signal peptide(feature SIGNALP is 15), no pattern starting with a P and followed within three residues by a basic four-residue segment containing K or R residues(NUC2 is 85), no C-terminal farnesylation site(FARN is 73), it has a higher probability of being transmembrane protein(T location).

Rule T1 shows when protein has no more than one N-terminal residue cut(MIT1 is 1), more possibility to have transmembrane(TMS1 is 43), viable knockout mutation(KNOCKOUT is 56), no pattern starting with a P and followed within three residues by a basic four-residue segment containing K or R residues(NUC2 is 85), no C-terminal peroxisome import signal(POX1 is 89), it has a higher probability of being transmembrane protein(T location).

Rule E1 shows that when protein has more than one N-terminal residue cut(feature MIT1 is 2), has secretory signal peptide(feature SIGNALP is 16), has a SIG1 signal(SIG1 is 19,see Table 1), no pattern starting with a P and followed within three residues by a basic four-residue segment containing K or R residues(NUC2 is 85), no C-terminal peroxisome import signal(POX1 is 89), it has a higher probability of being in E location.

In accordance with this analysis, we found that GLYC and SIGNALP features were informative to the location N, because N2 and N3 rules both include GLYC and SIGNALP information. Meanwhile, it could be inferred that proteins in N compartment were inclined to be of no secretory signal peptide and have more GLYC sites. For M and E compartment, MIT1 and SIGNALP were important features, which were both included in rules M1 and E1. M1 and E1 in that they both possessed the feature of having more than one N-terminal residue cut, and they differed in that the E compartment contained secretory signal peptide while the M compartment did not. Compartments C and T were similar in that no more than one N-terminal residue was cut and differed in that proteins in location C were inclined to have higher absolute mRNA expression. All these provided some clues for inferring the influence of features to the localizations of yeast proteins.

**Figure 1:**
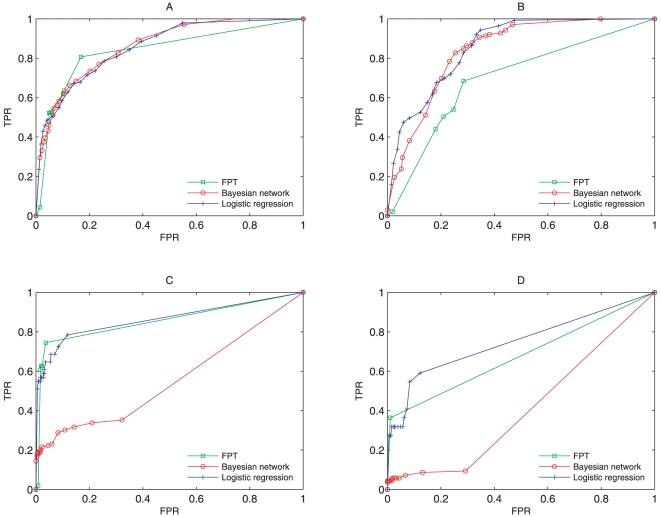
ROC curve comparisons of testing sample for 4 different compartments for 3 different methods. A,B,C,D show the comparison results separately for C, N, M, E compartment.

We predicted protein localization for training and testing data sets using rules obtained above. We evaluated the number of true/false positives predictions in the testing set and calculated Receiving Operator Characteristic (ROC) [18], which gives the quantitative measure of how good the discrimination is in identifying the protein localization. In Figure 1, green lines show the ROC curves of predicting C,N,M,E four locations using mFPT method. We can see that mFPT has its ROC curve climbing rapidly towards upper left hand corner of the graph, revealing a good prediction performance.

### Comparisons of Different Methods

In order to make comparisons to other data mining methods, we performed the prediction of protein localization using Bayesian network approach(BN) [17], [33]–[35], logistic regression method, and SNB(simple naive Bayesian classifier) [1] method separately. Figure 1A shows the comparisons of results of ROC curve for 3 different methods for C location for testing samples. Figure 1B, Figure 1C and Figure 1D show the comparing results of ROC curve for testing samples for the different methods for the N, M, E locations respectively.

From Figure 1A, we could not tell which one of three methods performed best because there were cross parts among their ROC curves. From Figure 1C and Figure 1D, we could see that the mFPT and the logistic regression methods performed better than the Bayesian network method, and the mFPT approach was moderately better than logistic regression. Only in Figure 1B, mFPT did not perform better than the other two methods. However, this did not affect the fact that mFPT gave the best overall prediction among these methods, which was also reflected in the next part about comparisons of correct prediction rate.

**Figure 2:**
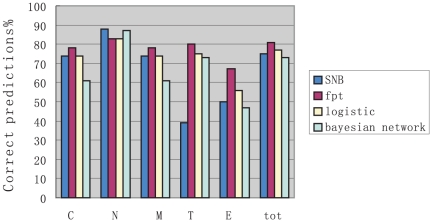
Comparisons of correct prediction rate for four methods.

As mentioned above, we also compared the correct prediction rate of four different methods. Figure 2 tabulates the comparison of prediction results per compartment as well as cumulatively for the various methods taken into consideration. It shows that mFPT reached a prediction accuracy of 81%, higher than SNB's 75%,logistic regression's 77%, and Bayesian network's 73%, and thus was the best performer of the methods.

**Figure 3:**
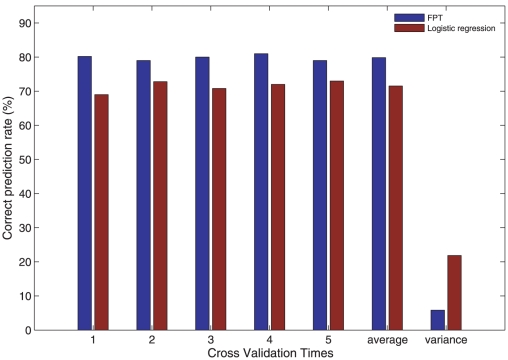
Comparisons of correct prediction rate using five fold cross validation test for FPT and logistic regression method. The variances are enlarged to 1000 times to see the comparison clearly.

In addition, we used cross validation (5-fold) to further validate the performance of FPT. In consideration of computing time cost, we just did cross validation to FPT and the logistic regression method that demonstrated the most similar performance. To implement five fold cross validation, we randomly divided 1342 samples into five subsets, each containing separately about 280 proteins. Then we predicted the localization of the proteins in each subset after training the system on the proteins in the remaining four subsets. Figure 3 shows the comparison results of the five fold cross validation test for FPT and logistic regression. We can see that FPT acquired 79% average correct prediction rate for testing samples, outperforming logistic regression (72%). The corresponding variances from the average are also compared after a thousand-fold enlargement. We found that the variance of performance of FPT is less than that of logistic regression. This showed that FPT is more robust and powerful method than logistic regression.

### Predict 4700 Yeast Proteins with Unknown Localization

**Figure 4:**
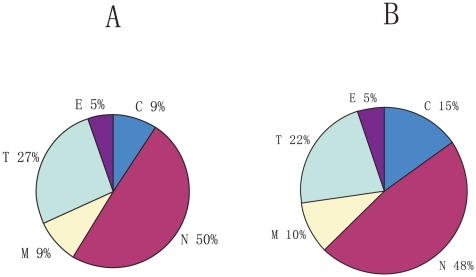
Prediction of localization for the entire 6042 yeast proteins.

After training and testing, we used our system to attempt to place the 4700 yeast proteins that did not have a known localization [1]. To determine the locations of these 4700 yeast proteins(we call this set the Unknown-4700), we trained the feature values on the Localized-1342 set, and used the rules from Localized-1342 to predict the overall compartment population for the Unknown-4700 proteins. Setting 0.5 as the minimum hit-rate cut, we retrieved 3787 proteins with predicted locations for the total 4700 ones using forementioned 142 rules. Figure 4A shows prediction results for Unknown-4700(see the detailed prediction results in File S2).

In order to explore more deeply what we found using mFPT, we set the minimum hit-rate as 0.65 to predict the Unknown-4700 again. In this way, we increased the correct prediction rate, and therefore retreived fewer proteins with predicted location for the 4700 proteins compared with the results when we used 0.5 as the minimum hit-rate threshold. This time we got 6 rules as shown in Table 4, and only predicted 1261 proteins using these rules. We then compared the different parts of predictions of SNB and mFPT for Unknown-4700 and obtained 138 proteins as shown in Table S1. Meanwhile, we searched for these proteins in SGD(Saccharomyces Genome Database) and Swiss-Prot database to make comparisons and explore their localization information.

In Table S1 there are total 138 proteins, 17 of which (12%) have no annotated locations in SGD, 77 of which (56%) were correctly predicted by mFPT based on SGD or Swiss-Prot. The percentage of correct predictions for five locations are respectively 50%,31%,62%,95%,29%,(C,N,M,T,E).

We found that for the N and E compartments of Unknown-4700, the proteins mFPT predict correctly are only 31% and 29%, which was potentially due to there being only 1342 proteins with limited features as the training samples for predicting five locations of preteins, almopst certainly did not reflect the totality of characteristics of the yeast proteome. We believe that with more features and proteins included, mFPT coud have outputted a greater number of predictions.

For the Unknown-4700, we predicted some new tentative localizations for 17 proteins that currently do not have any associated localization, whose annotations are described in detail in the discussion section. These predictions can serve as putative annotations and should be experimentally validated. Meanwhile, they provide preliminary clues for experimentalists.

Finally, we integrated results of prediction of both the Unknown-4700 dataset and Localize-1342 dataset, and acquired the predicting results of the entire yeast genome (6042 proteins), which is shown in Figure 4B. From Figure 4B, we could see that nucleus proteins occuppied the largest share of the predictions for localization, probably because that the training data were strongly biased towards nucleus proteins [1].

We also compared our prediction results with a 0.65 minimum hit-rate threshold against eSLDB(eukaryotic subcellular localization database) [14], and related prediction by others on protein localization (see SI Text and File S3).

## Discussion

From Table S1, we could find that for C location, there are 2 proteins newly predicted. YHR020W colocalizes with ribosome [36], and should belong to cytoplasm location, in that ribosome proteins occupy 7.5% of cytoplasm location [3]. YNL327W belongs to protein of cellular bud or cell wall [29], [30].

For the N location predicted by mFPT, there were 11 proteins newly predicted. YLR149C is a putative protein of unknown function, over expression of which causes a cell cycle delay or arrest [37]. 12.3% proteins of nucleus location are related with cell cycle and mitosis [3], so, it might be one nucleus protein. YGL215W is a Cyclin-like protein that interacts with Pho85p. YOR043W is a protein that along with its binding partner Psr1p, regulates growth during the diauxic shift, and also serves as a negative regulator of G1 cyclin expression. YPL219W is a Cyclin, interacts with Pho85p cyclin-dependent kinase (Cdk) to phosphorylate, and regulates glycogen synthase, and YBL047C is cellular bud neck proteins. These four proteins are all related with the cell cycle and might be classified to the nuclear compartment. The gene of YHR219W is located in the telomere region on the right arm of chromosome VIII, and should also belong to nuclear compartment. YPR204W affects DNA helicase activity [38], and therefore is located in the nuclear compartment. YEL062W is a protein with a possible role in regulating expression of the nitrogen permeases, and YNL229C is a nitrogen catabolite repression transcriptional regulator that acts by inhibition of GLN3 transcription when nitrogen is in abundance. These two proteins are both associated with transcription, and therefore might be classified to nuclear compartment. YPL054W is a Zinc-finger protein of unknown function. YIL151C is a putative protein of unknown function, predicted to contain a PINc (PilT N terminus) domain. For these two proteins, there is no evidence that would lead one to assign them to the nuclear compartment.

For M location predicted by mFPT, there was only 1 protein newly predicted. The gene of YDL183C is on the right arm of chromosome IV [39]. We looked up this protein in the SGD and YRc but did not find any related evidence to make it localized to M compartment.

mFPT proved to be very effective for predicting T localization as there was only one protein YOL007C newly predicted. SGD showed that YOL007C localizes to the other side of the bud neck and the vacuole, and according to TMHMM, a program of predicting transmembrane helices of proteins [40], it might have one TM-helix, which would be consistent with our prediction.

For E location predicted by mFPT, there were only 2 proteins newly predicted. YJL193W is putative protein of unknown function, predicted to encode a triose phosphate transporter subfamily member based on phylogenetic analysis [41], and is related with transport, and might be classified to E location. YNL322C is Cell wall glycoprotein involved in beta-glucan assembly, and should belong to E location.

### Conclusion

In conclusion, we have applied a mFPT approach to predict protein localization integrating all kinds of genomic features. The ROC curve of prediction results for Localizaed-1342 dataset indicated that this approach performs better than logistic regression, Bayesian network and other methods which are commonly used. We also made this prediction for the Unknown-4700 dataset using FPT. When we chose 0.65 as the minimum hit-rate, we got 1261 proteins with predicted locations. Comparing the different part of predictions of SNB and FPT for the Unknown 4700, we acquired 138 proteins, among which 77(56%) were predicted correctly by FPT compared with SGD or Swiss-Prot or Mips. We also provided new tentative localizations for 17 proteins that currently do not have any associated localization. These predictions can serve as leads for future experimentation.

It stands to reason that the addition of more data and better features to our method would lead to better distinction of localization among the compartments as well as to greater accuracy of prediction. Also, it is anticipated that this approach could be used to uncover protein interactions and localizations in other organisms and may also be applied to studies of gene networks. As a general tool, mFPT has the potential to be applied for not only biology but other areas in science and industry where large data and information mining are required.

## Materials and Methods

### Method of Frequent-pattern Mining

FP(frequent patterns) mining is a very important approach in the field of data mining used to extract significant and potentially useful patterns from some large database. And the information and knowledge gained can be applied to a diverse body of fields: from market analysis to fraud detection, customer retention, production control, as well as to other avenues of science inquiry [24].

First, we check Frequent-pattern mining [21], [22]. Let 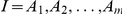 be a set of items, and a transaction database 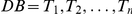, where 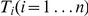 is a transaction containing a set of items in 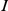. The support (or occurrence frequency) of a pattern 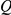, where 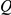 is a set of items, is the number of transactions containing 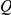 in 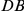. Pattern 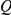 is frequent if 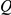
's support is bigger than a predefined minimum support threshold, 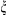. Given a transaction database DB and a minimum support threshold 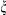, finding the complete set of frequent patters is called the frequent-pattern mining problem. [21], [22].

Mining frequent patterns in transaction databases, has been studied popularly in data mining. Most previous studies on frequent pattern mining adopt the Apriori algorithm [42]. This method has bottlenecks, which are the huge candidate sets and multiple scans of the entire database with high computational costs.

FPT method discovers frequent patterns in transactional databases by FP-growth arithmetic. FP-growth [22] first performs a frequent item-based databases projection to the large database and then a compact data structure, FP-tree, is constructed, which is condensed, but complete for frequent pattern mining. Therefore, mining database problem is transformed into mining one compact tree. The FPT approach has several advantages compared with some representative frequent-pattern mining methods for data mining: It alleviates the multi-scan problem and improves the candidate pattern generation; it is faster than Apriori and performs better than the tree projection algorithm [43] and it shows advantages particularly when the data set contain many patterns or the frequent patterns are long [21], [22].

A frequent-pattern tree (or FP-tree in short) is a tree structure and it can be designed as follows [21].

1. It comprises one root labeled as "null", a set of item-prefix subtrees as the children of the root, and a frequent-item-header table.
2. Each node in the item-prefix subtree includes three fields: item-name, count, and node-link, where item-name denotes which item this node represents, count denotes the number of transactions represented by the portion of the path reaching this node, and node-link links to the next node in the FP-tree carrying the same item-name. Node-link is null if there is no next node.
3. Each entry of the frequent-item-header table is made up of two fields, (1) item-name and (2) head of node-link (a pointer pointing to the first node in the FP-tree carrying the item-name).

According the design principle, after scanning all the transactions, the FP-tree could be constructed. First, a scan of 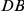 produces a list of frequent items, such as 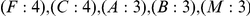 (here 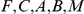 represent items, and the number after ":" indicates the support), and items in this list are ordered in frequency-descending order. Second, the root of a tree labeled with "null" is created.

Table 5 is an example for the transaction database, 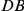, in which the minimum support threshold is 3. The FP-tree is constructed as follows by scanning the transaction database 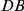 twice.

1. The scan of the first transaction construct the first branch of the tree: 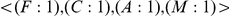. Here the frequent items in the transaction are listed in terms of the order in the list of frequent items.
2. For the second transaction, its (ordered) frequent item list 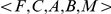 shares a common prefix 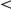 F, C, A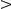 with the existing path 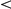
F, C, A, M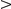, so the count of each node along the prefix is increased by 1, and one new node 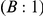 is created and linked as a child of 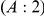 and another new node 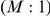 is created and linked as the child of 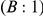.
3. For the third transaction, 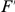
s count is incremented by 1, and a new node 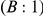 is created and linked as a child of (F:3), because its frequent item list 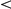 F, B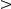 shares only the node 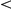 F 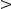 with the F -prefix subtree.
4. Scanning the fourth transaction leads to the construction of the second branch of the tree, 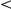
(C:1), (B:1)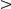.
5. For the last transaction, its frequent item list 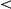
F, C,A,M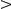 is same with the first one. Hence, the path is shared with the count of each node along the path incremented by 1.

**Table 5:** The FP-tree in Example 1.

Tid	Items bought	(Ordered) frequent items
1	F, A, C, D, G, I, M	F, C, A, M
2	A, B, C, F, L, M, O	F, C, A, B, M
3	B, F, H, J, O	F, B
4	B, C, K, S	C, B
5	A, F, C, E, L, M, N	F, C, A, M

**Figure 5:**
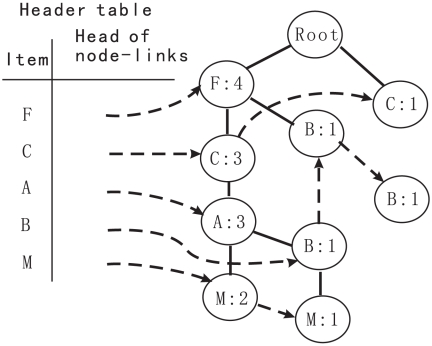
The FP-tree in Example 1.

In order to promote tree traversal, an item header table is built in which each item points to its first occurrence in the tree via a node-link. Nodes with the same item-name are linked in sequence by such node-links. After scanning all the transactions, the tree, accompanied by the related node-links, are shown in Figure 5.

### Logistic Regression

Features are denoted by 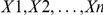, and the locations are denoted by variable 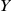. Five files of features are constructed respectively for 5 location prediction. For every individual location prediction, the output variable 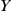 is set to binary format. 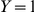 represents protein belong to this location, while 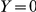 denotes not. The logistic model is of the form 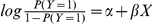, where the vector 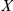 consists of 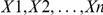, and 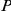 represents probability [18]. We use SAS(SAS 8.2) software to implement the logistic regression process.

## Supporting Information

File S1 Rules for the predictions of 4700 proteins with the hit-rate threshold cut 0.5. The first two columns represent respectively the hit-rate and hit-number of the FPT rules. The last column represents prediction locations, and between them are FPT rules.
(0.00 MB TXT)

File S2 Predictions for the entire yeast proteome with the hit-rate threshold cut 0.5. There are 3,787 proteins with predicted locations by FPT using 142 rules (see the rules in File S1). We compared these prediction results against SNB method. The first column (ID) represents proteins. The second column (SNB) represents prediction results of SNB. The third column (FPT) represents the prediction results of FPT at 0.5 hit-rate threshold, and the last column(hit-rate) represents the hit-rate of rules that FPT use.
(0.07 MB TXT)

File S3 Prediction difference between our work and eSLDB. There are 504 proteins predicted differently by our FPT method and eSLDB among 1,267 proteins we acquired at 0.65 hit-rate threshold cut, 98 proteins of which we randomly selected and looked up in both SGD and YRC (http://images.yeastrc.org/). In File S3, the first column (ID) represents proteins. The second column (FPT065) represents prediction results of FPT at 0.65 hit-rate threshold. The third column (HITRATE) represents the hit-rate of rules that FPT use. The fourth column (eSLDB) represents prediction results of eSLDB. The fifth column (locations) denotes the locations recorded in Database, with "un" denoting that the there is no explicit description about the localization of this protein from the three databases. And the sixth and seventh columns denote separately the source of database and descriptions in the databases (SGD or YRC).
(0.01 MB TXT)

Table S1 Differences in prediction results of Unknown 4700 between FPT and SNB. Our FPT uses 0.65 as hit-rate threshold, and there are totally 138 proteins in this table, which are predicted using the rules in Table 4. N represents nucleus, M for mitochondria, C for cytoplasm, T for membrane, and E for endoplasmic reticulum or extracellular. Here, the database includes SGD (Saccharomyces Genome Database), Swiss-Prot, and Mips. About the location from database column, the default is from SGD, and the items from Mips and Swiss-Prot have been indicated. Unknown denotes that there is no explicit description about the localization of this protein from the three databases. For Swiss-Prot, the term "Potential" indicates that there are some logical or conclusive evidences that the given annotation could apply. This nonexperimental qualifier is often used to present the results from protein sequence analysis tools, which are only annotated, if the results make sense in the context of a given protein. The term "Probable" is stronger than "Potential", and there must be at least some experimental evidence that indicates that the given information is expected to be found in the natural environment of a protein.
(0.05 MB PDF)

Text S1 Supplementary results of comparisons between FPT and eSLDB.
(0.03 MB PDF)
