# Supplementary material for: An FPT Approach for Predicting Protein Localization from Yeast Genomic Data
Source: PLoS One. 2011 Jan 19;6(1):e14449. doi: 10.1371/journal.pone.0014449 (PMC3023707; doi:10.1371/journal.pone.0014449)
Supplement: Table S1 — Differences in prediction results of Unknown 4700 between FPT and SNB. Our FPT uses 0.65 as hit-rate threshold, and there are totally 138 proteins in this table, which are predicted using the rules in Table 4. N represents nucleus, M for mitochondria, C for cytoplasm, T for membrane, and E for endoplasmic reticulum or extracellular. Here, the database includes SGD (Saccharomyces Genome Database), Swiss-Prot, and Mips. About the location from database column, the default is from SGD, and the items from Mips and Swiss-Prot have been indicated. Unknown denotes that there is no explicit description about the localization of this protein from the three databases. For Swiss-Prot, the term "Potential" indicates that there are some logical or conclusive evidences that the given annotation could apply. This nonexperimental qualifier is often used to present the results from protein sequence analysis tools, which are only annotated, if the results make sense in the context of a given protein. The term "Probable" is stronger than "Potential", and there must be at least some experimental evidence that indicates that the given information is expected to be found in the natural environment of a protein. (0.05 MB PDF) [file pone.0014449.s004.pdf]

Table S 1: **Differences of prediction results of Unknown 4700 between FPT and SNB.** FPT use 0.65 as hit-rate threshold, and there are totally 138 proteins in this table, which are predicted using the rules in Table 4. N represents nucleus, M for mitochondria, C for cytoplasm, T for membrane, and E for endoplasmic reticulum or extracellular. Here database include SGD(Saccharomyces Genome Database),Swiss-Prot, and Mips. About the column of location from database, the default is from SGD, and the items from Mips and Swiss-Prot have been indicated. Unknown denotes that the there are no explicit description about the localization of this protein from the three database. For Swiss-Prot, the term 'Potential' indicates that there is some logical or conclusive evidence that the given annotation could apply. This non-experimental qualifier is often used to present the results from protein sequence analysis tools, which are only annotated, if the result makes sense in the context of a given protein. The term 'Probable' is stronger than the qualifier 'Potential' and there must be at least some experimental evidence, which indicates, that the given information is expected to be found in the natural environment of a protein.

Systematic Name	SNB	FPT	rule	location from database	description from databse
YOR007C	T	C	C1	C	Glutamine-rich cytoplasmic protein of unknown function
YOR247W	E	C	C1	C	Mannoprotein that exhibits a tight association with the cell wall
YAL035W	N	C	C1	C M	GTPase,required for general translation initiation by promoting Met-tRNAiMet binding to ribosomes and ribosomal subunit joining;
YKL182W	N	C	C1	C M	Beta subunit of fatty acid synthetase
YPR149W	E	C	C1	C M	Protein of unknown function; contains transmembrane domains
YJL130C	T	C	C1	C M T	This protein is a "fusion" protein encoding three enzymatic activities of the pyrimidine pathway
YKL117W	T	C	C1	C N	Acts as a co-chaperone
YPR062W	T	C	C1	C N	Cytosine deaminase, zinc metalloenzyme that catalyzes the hydrolytic deamination of cytosine to urac
YDL126C	N	C	C1	C N T	ATPase in ER, nuclear membrane and cytosol with homology to mammalian p97
YLR421C	T	C	C1	C N(Swiss-Prot)	Subunit of the 19S regulatory particle of the 26S proteasome lid
YAR009C	N	C	C1	C N(Swiss-Prot)	Cytoplasm, Nucleus
YDR172W	N	C	C1	C(Swiss-Prot)	Involved in translation termination
YJR085C	T	C	C1	M T(Swiss-Prot)	Mitochondrion, Membrane; Multi-pass membrane protein(Potential)

Continued...

Systematic Name	SNB	FPT	rule	location from database	description from databse
YPR028W	T	C	C1	T	Membrane protein that interacts with Yip1p to mediate membrane traffic;
YPR165W	T	C	C1	T	GTP-binding protein of the rho subfamily of Ras-like proteins
YDR233C	T	C	C1	T(Swiss-Prot)	Endoplasmic reticulum membrane,Multi-pass membrane protein
YHR026W	T	C	C1	T(Swiss-Prot)	Membrane; Multi-pass membrane protein
YIL043C	T	C	C1	T(Swiss-Prot)	Endoplasmic reticulum membrane; Single-pass membrane protein. Mitochondrion outer membrane;
YHR020W	N	C	C1	unknown	Protein of unknown function that may interact with ribosomes
YNL190W	T	C	C1	E(Mips)	Cell wall protein of unknown function
YBR162C	E	C	C1	N E(Mips)	Covalently-bound cell wall protein of unknown function
YEL040W	E	C	C1	E	Cell wall protein that functions in the transfer of chitin to beta(1-6)glucan
YGR279C	E	C	C1	E	Glucanases possibly play a role in cell expansion during growth,in cell-cell fusion during mating
YNL327W	E	C	C1	unknown	Glycosylphosphatidylinositol (GPI)-anchored cell wall endoglucanase required for proper cell separat
YAL007C	T	E	E1	E	Protein that forms a heterotrimeric complex with Erp1p, Emp24p, and Erv25p
YIR022W	T	E	E1	E	18kDa catalytic subunit of the Signal Peptidase Complex
YNL219C	T	E	E1	E	endoplasmic reticulum
YNL194C	T	E	E1	E C	Integral membrane protein required for sporulation and plasma membrane sphingolipid content
YMR035W	M	E	E1	M	Catalytic subunit of the mitochondrial inner membrane peptidase complex
YKL046C	T	E	E1	T	Putative mannosidase, GPI-anchored membrane protein required for cell wall biosynthesis in bud forma
YKL220C	T	E	E1	T	plasma membrane
YDR420W	T	E	E1	T(Swiss-Prot)	Membrane; Single-pass type I membrane protein (Probable).
YHR211W	T	E	E1	T(Swiss-Prot)	Cell membrane; Lipid-anchor, GPI-anchor (Potential).
YMR149W	T	E	E1	T(Swiss-Prot)	Endoplasmic reticulum membrane; Multi-pass membrane protein (Probable).
YHL026C	T	E	E1	T	Membrane; Multi-pass membrane protein (Potential)
YJL193W	T	E	E1	unknown	Putative protein of unknown function

Continued...

Systematic Name	SNB	FPT	rule	location from database	description from databse
YKL039W	T	E	E1	T(Swiss-Prot)	Protein of unknown function, copurifies with late Golgi vesicles containing the v-SNARE Tlg2p
YNL322C	T	E	E1	unknown	Cell wall glycoprotein involved in beta-glucan assembly
YJL001W	T	M	M1	C N	The proteasome degrades poly-ubiquitinated proteins in the cytoplasm and in the nucleus
YDL171C	T	M	M1	M	GLuTamate synthase
YER017C	T	M	M1	M	Component of the mitochondrial inner membrane
YKL141W	T	M	M1	M	mitochondrial respiratory chain complex II
YMR301C	T	M	M1	M	Mitochondrial inner membrane ATP-binding cassette (ABC) transporter
YNR041C	T	M	M1	M	Para hydroxybenzoate
YML042W	E	M	M1	M	Carnitine acetyl-CoA transferase present in both mitochondria and peroxisomes
YMR307W	E	M	M1	M T	Beta-1,3-glucanosyltransferase, required for cell wall assembly;
YPR183W	T	M	M1	M T E	Dolichol Phosphate Mannose synthase
YGL065C	T	M	M1	T(Swiss-Prot)	Endoplasmic reticulum membrane; Multi-pass membrane protein.
YLR220W	T	M	M1	T(Swiss-Prot)	Putative vacuolar Fe2+/Mn2+ transporter
YDL183C	T	M	M1	unknown	Putative protein of unknown function;
YOL052C	T	M	M1	N C	S-adenosylmethionine decarboxylase is essential for normal growth, sporulation, maintenance of ds-RN
YDL167C	C	N	N2	C	Protein of unknown function, rich in asparagine residues
YOL061W	C	N	N3	C	5-phospho-ribosyl-1(alpha)-pyrophosphate synthetase, synthesizes PRPP, which is required for nucleot
YDR127W	T	N	N2	C	Pentafunctional arom protein
YGL206C	T	N	N2	C	Cytoplasmic vesicle membrane; Peripheral membrane protein
YDL189W	T	N	N3	C	Protein of unknown function
YLR143W	T	N	N3	C	Putative protein of unknown function;
YOL136C	T	N	N3	C	fructose 2,6-bisphosphate metabolic process
YOR035C	T	N	N3	C	Swi5p-dependent HO Expression
YOL141W	T	N	N3	C M	tRNA methyltransferase required for the synthesis of wybutosine
YBR238C	C	N	N3	C M T	Mitochondrial membrane protein with similarity to Rmd9p
YGL161C	T	N	N3	E	Protein that interacts with Rab GTPases, localized to late Golgi vesicles
YMR006C	T	N	N2	E T	Phospholipase B (lysophospholipase) involved in phospholipid metabolism

Continued…

Systematic Name	SNB	FPT	rule	location from database	description from databse
YGL129C	M	N	N3	M	Mitochondrial ribosomal protein of the small subunit
YDR430C	T	N	N3	M	Lysine-specific metalloprotease of the mitochondrial intermembrane space
YKL195W	T	N	N3	M	Mitochondrion inner membrane; Single-pass type II membrane protein
YLR454W	E	N	N2	M	Putative protein of unknown function
YNL121C	T	N	N3	M T(Swiss-Prot)	Mitochondrion outer membrane; Single-pass membrane protein
YJL089W	C	N	N2	N	C6 zinc cluster transcriptional activator that binds to the carbon source-responsive element (CSRE)
YBR186W	C	N	N3	N	Nucleolar component of the pachytene checkpoint,
YER110C	C	N	N3	N C	localizes to the nuclear pore, nucleus, and cytoplasm
YJL157C	C	N	N3	N C	Cyclin-dependent kinase inhibitor that mediates cell cycle arrest in response to pheromone
YPL012W	C	N	N3	N C	Protein required for export of the ribosomal subunits
YEL007W	T	N	N2	N C	Putative protein with sequence similarity to S. pombe gti1+ (gluconate transport inducer 1)
YLR176C	T	N	N2	N C	Major transcriptional repressor of DNA-damage-regulated genes
YBL058W	T	N	N3	N C	Suppressor of High-copy PP1
YBR281C	T	N	N3	N C	Probable di- and tri-peptidase
YPR118W	T	N	N3	N C	5'-methylthioribose-1-phosphate isomerase;
YMR129W	C	N	N3	N M	Nucleus, nuclear pore complex. Nucleus membrane; Single-pass type II membrane protein
YOR206W	C	N	N3	N M	Protein that forms a nucleolar complex with Mak21p that binds to 90S and 66S preribosomes,
YNL008C	T	N	N3	N T	Putative integral membrane E3 ubiquitin ligase
YML006C	T	N	N2	T	plasma membrane
YBL063W	C	N	N2	N	Kinesin-related motor protein required for mitotic spindle assembly and chromosome segregation
YLR149C	C	N	N2	unknown	Putative protein of unknown function
YPL054W	C	N	N3	unknown	Zinc-finger protein of unknown function
YBL047C	T	N	N2	unknown	cellular bud neck
YHR219W	T	N	N2	unknown	Putative protein of unknown function with similarity to helicases;

Continued…

Systematic Name	SNB	FPT	rule	location from database	description from databse
YIL151C	T	N	N2	unknown	Putative protein of unknown function, predicted to contain a PINc (PilT N terminus) domain
YPR204W	T	N	N2	unknown	Helicase-like protein encoded within the telomeric Y' element
YEL062W	T	N	N3	unknown	Protein with a possible role in regulating expression of nitrogen permeases
YGL215W	T	N	N3	unknown	Cyclin-like protein that interacts with Pho85p
YJR039W	T	N	N3	M	Putative protein of unknown function
YLR389C	T	N	N3	M	Involved in a-factor processing.
YNL229C	T	N	N3	unknown	Nitrogen catabolite repression transcriptional regulator that acts by inhibition of GLN3 transcripti
YOR043W	T	N	N3	unknown	Plays a role in the coordination of growth and proliferation
YPL219W	T	N	N3	unknown	cyclin-dependent protein kinase holoenzyme complex
YKL187C	E	T	T1	M	Putative protein of unknown function; the authentic, non-tagged protein is detected in a phosphoryla
YBR069C	C	T	T1	T	plasma membrane
YBR132C	C	T	T1	T	endoplasmic reticulum membrane,vacuolar membrane
YBR241C	C	T	T1	T	Putative transporter, member of the sugar porter family; green fluorescent protein (GFP)-fusion prot
YDR536W	C	T	T1	T	Glycerol proton symporter of the plasma membrane
YBR187W	E	T	T1	T	fungal-type vacuole
YGR014W	E	T	T1	T	integral to plasma membrane
YOL019W	E	T	T1	T	Protein of unknown function; green fluorescent protein (GFP)-fusion protein localizes to the cell pe
YMR058W	E	T	T1	T E(Swiss-Prot)	Cell membrane; Single-pass type I membrane protein; Extracellular side
YDR033W	C	T	T1	T M	Protein that localizes primarily to the plasma membrane, also found at the nuclear envelope; the aut
YDR384C	C	T	T1	T M	Plasma membrane protein, regulation pattern suggests a possible role in export of ammonia from the c
YDR508C	C	T	T1	T M	GlutamiNe Permease
YLR342W	C	T	T1	T M(Swiss-Prot)	Mitochondrion. Cell membrane; Multi-pass membrane protein
YBR266C	C	T	T1	T(Swiss-Prot)	Membrane; Multi-pass membrane protein.

Continued...

Systematic Name	SNB	FPT	rule	location from database	description from databse
YER072W	C	T	T1	T(Swiss-Prot)	Vacuole membrane; Multi-pass membrane protein
YFL026W	C	T	T1	T(Swiss-Prot)	Membrane; Multi-pass membrane protein
YGR055W	C	T	T1	T(Swiss-Prot)	Membrane; Multi-pass membrane protein
YGR160W	C	T	T1	T(Swiss-Prot)	Membrane; Single-pass membrane protein
YJL196C	C	T	T1	T(Swiss-Prot)	Membrane; Multi-pass membrane protein
YJR143C	C	T	T1	T(Swiss-Prot)	Endoplasmic reticulum membrane; Multi-pass membrane protein
YLR056W	C	T	T1	T(Swiss-Prot)	Endoplasmic reticulum membrane; Multi-pass membrane protein
YML123C	C	T	T1	T(Swiss-Prot)	Membrane; Multi-pass membrane protein.
YMR011W	C	T	T1	T(Swiss-Prot)	Membrane; Multi-pass membrane protein
YNL065W	C	T	T1	T(Swiss-Prot)	Membrane; Multi-pass membrane protein.
YNL174W	C	T	T1	T(Swiss-Prot)	Membrane; Multi-pass membrane protein
YOR153W	C	T	T1	T(Swiss-Prot)	Cell membrane; Multi-pass membrane protein
YPL019C	C	T	T1	T(Swiss-Prot)	Vacuole membrane; Multi-pass membrane protein
YJL129C	N	T	T1	T(Swiss-Prot)	Membrane; Multi-pass membrane protein
YER001W	E	T	T1	T(Swiss-Prot)	Golgi apparatus membrane; Single-pass type II membrane protein
YHR149C	E	T	T1	T(Swiss-Prot)	Membrane; Single-pass membrane protein
YIL037C	E	T	T1	T(Swiss-Prot)	Membrane; Multi-pass membrane protein
YIL173W	E	T	T1	T(Swiss-Prot)	Endosome membrane; Single-pass membrane protein
YJL051W	E	T	T1	T(Swiss-Prot)	Membrane; Multi-pass membrane protein.
YKL178C	E	T	T1	T(Swiss-Prot)	Membrane; Multi-pass membrane protein
YLR084C	E	T	T1	T(Swiss-Prot)	Cell membrane; Single-pass type I membrane protein
YLR343W	E	T	T1	T(Swiss-Prot)	Cell membrane; Lipid-anchor, GPI-anchor
YLR413W	E	T	T1	T(Swiss-Prot)	Cell membrane; Multi-pass membrane protein
YNL279W	E	T	T1	T(Swiss-Prot)	Cell membrane; Multi-pass membrane protein
YNL283C	E	T	T1	T(Swiss-Prot)	Membrane; Single-pass membrane protein
YAL018C	C	T	T1	T(Swiss-Prot)	Membrane; Multi-pass membrane protein
YAL023C	C	T	T1	T(Swiss-Prot)	Endoplasmic reticulum membrane; Multi-pass membrane protein
YOL007C	E	T	T1	unknown	Protein of unknown function; green fluorescent protein (GFP)-fusion protein localizes to themother
