# Supplementary material for: An FPT Approach for Predicting Protein Localization from Yeast Genomic Data
Source: PLoS One. 2011 Jan 19;6(1):e14449. doi: 10.1371/journal.pone.0014449 (PMC3023707; doi:10.1371/journal.pone.0014449)
Supplement: Text S1 — Supplementary results of comparisons between FPT and eSLDB. (0.03 MB PDF) [file pone.0014449.s005.pdf]

Supporting Information: A FPT Approach for Predicting Protein Localization from Yeast Genomic Data

Jin Wang[1,2,*], Chunhe Li[1,3], Erkang Wang[1,*],Xidi Wang[4]
1 State Key Laboratory of Electroanalytical Chemistry,Changchun Institute of Applied Chemistry, Chinese Academy of Sciences, Changchun, Jilin, China
2 Department of Chemistry, Physics, and Applied Mathematics, State University of New York at Stony Brook, Stony Brook, NY, United States of America
3 Graduate School of the Chinese Academy of Sciences,Beijing, China
4 Citibank, Paulista 1111, Sao Paulo, 01311-920, Brazil
∗ E-mail: jin.wang.1@stonybrook.edu, ekwang@ciac.jl.cn

Supplementary results

Comparisons with eSLDB

We also compared our results with one of other prediction results [1], which has constructed Eukaryotic Subcellular Localization DataBase(eSLDB), annotating subcellular localization of eukaryotic proteomes. We downloaded their prediction results for Saccharomyces cerevisiae, 6680 proteins with locations predicted, and made comparisons with our 1261 prediction results at 0.65 minimum hit-rate threshold. 1251 proteins appear both in our results and theirs, among which 747 proteins(60%) have the same predicted locations and 504 proteins(40%) have different predicted locations. In these 504 proteins with different predicted locations, we randomly selected 50 proteins three times. Every time we looked up the subcellular localization of these 50 proteins in both SGD and YRC [2,3], and found that on average 19(38%) of 50 proteins are predicted correctly by FPT in terms of SGD or YRC, whereas the corresponding correctly number of correctly predicted is 12(24%) for eSLDB. The number of unknown locations in both SGD or YRC is 13(26%), and the number of proteins wrongly predicted by both methods is 7(14%). This shows that our method predicts more accurately when we set 0.65 as the hit-rate threshold, and could be applied more deeply to explore proteins without known annotations in current database or experiments, although we can only give 1261 predictions for the Unknown-4700 dataset. In File S3, we show the detailed prediction results of 504 proteins for both FPT and eSLDB, 150 of which we give randomly chosen localization information from SGD or YRC.

References

1. Pierleoni A, Martelli PL, Fariselli P, Casadio R (2006) esldb: eukaryotic subcellular localization database. *NUCLEIC ACIDS RES* 35: D208-D212.

2. Huh WK, Falvo JV, Gerke LC, Carroll AS, Howson RW, et al. (2003) Global analysis of protein localization in budding yeast. *Nature* 425: 686-691. 30.

3. Riffle M, Davis T (2010) The yeast resource center public image repository: A large database of uorescence microscopy images. *BMC Bioinfromatics* 11: 263.
